# Clinical significance of hyperbilirubinemia in the CASTLE study

**DOI:** 10.1186/1758-2652-13-S4-P93

**Published:** 2010-11-08

**Authors:** J Uy, W Hu, V Wirtz, T Hosey, D Butcher, D McGrath, A Farajallah

**Affiliations:** 1 Bristol-Myers Squibb Research & Development, 777 Scudders Mill Rd, Plainsboro, NJ, USA; 2 Bristol-Myers Squibb Research & Development, Wallingford, CT, USA; 3 Bristol-Myers Squibb Research & Development, Rueil-Malmaison, France

## Purpose of the study

While unconjugated hyperbilirubinemia is associated with the use of ritonavir-boosted atazanavir (ATV/r), the nature of the hyperbilirubinemia over time and its clinical significance has not been well-characterized in controlled studies. The purpose of this study is to describe the patterns and clinical significance of hyperbilirubinemia in patients treated with ATV/r in the CASTLE study.

## Methods

CASTLE was a randomized, 96-week study to assess the efficacy and safety of ATV/r vs. lopinavir/r, each with tenofovir/emtricitabine, in treatment-naïve patients. This analysis included only ATV/r patients. The proportions of patients with hyperbilirubinemia (grades 3-4 total bilirubin elevation) were tabulated for each study visit. The impact of hyperbilirubinemia on symptoms (jaundice or scleral icterus), ASL/ALT elevations, quality of life (MOS-HIV physical and mental summary scores), and adherence (MACS adherence questionnaire) were described.

## Summary of results

Although the proportion of patients with hyperbilirubinemia at any time throughout the study was 44%, the proportion of ATV/r patients with hyperbilirubinemia at any single visit was between 12.5% and 21.6%. Of patients with hyperbilirubinemia at any time, 11% had grades 2-4 treatment-related jaundice or scleral icterus at any time (0 of patients without hyperbilirubinemia), and 4% had grades 3-4 AST/ALT elevations at any time (3% of patients without hyperbilirubinemia). Quality of life and adherence in patients without and with hyperbilirubinemia. Table 1.

**Table 1:** 

		Patients without hyperbilirubinemia	Patients with hyperbilirubinemia
MOS-HIV Physical Summary Score Categories at Week 96			

	Improvement	76/138 (55%)	70/128 (55%)

	No change	35/138 (25%)	29/128 (23%)

	Worsening	27/138 (20%)	29/128 (23%)

MOS-HIV Mental Summary Score Categories at Week 96			

	Improvement	97/138 (70%)	92/128 (72%)

	No change	25/138 (18%)	18/128 (14%)

	Worsening	16/138 (12%)	18/128 (14%)

Adherence Through Week 96			

	To regimen	154/186 (83%)	147/176 (84%)

	To ATV	159/186 (85%)	153/176 (87%)

## Conclusions

Hyperbilirubinemia, while common in patients on ATV/r at any time through 96 weeks in the CASTLE study, was less frequent at any single time point and not associated with related symptoms in most patients. The presence of hyperbilirubinemia did not affect AST/ALT elevations, quality of life, or adherence. These data suggest that hyperbilirubinemia observed with ATV/r does not impact clinical outcomes.

